# Automated Detection and Grading of Renal Cell Carcinoma in Histopathological Images via Efficient Attention Transformer Network

**DOI:** 10.3390/medsci13040257

**Published:** 2025-11-01

**Authors:** Hissa Al-kuwari, Belqes Alshami, Aisha Al-Khinji, Adnan Haider, Muhammad Arsalan

**Affiliations:** 1 College of Medicine, Qatar University, Doha 2713, Qatar; ha2104524@qu.edu.qa (H.A.-k.); aalkhinji@qu.edu.qa (A.A.-K.); 2 College of Engineering, Qatar University, Doha 2713, Qatar; ba2002978@qu.edu.qa; 3 Translational Science Research Network Group, QU Health, Qatar University, Doha 2713, Qatar; 4 Metaverse R&D Center, Institute of Future Technology, Dongguk University, 30 Pildong-ro 1-gil, Jung-gu, Seoul 04620, Republic of Korea; adnanhaider@dgu.ac.kr

**Keywords:** renal cell carcinoma, histopathology, deep learning, efficientNet, vision transformer, medical image classification

## Abstract

**Background:** Renal Cell Carcinoma (RCC) is the most common type of kidney cancer and requires accurate histopathological grading for effective prognosis and treatment planning. However, manual grading is time-consuming, subjective, and susceptible to inter-observer variability. **Objective:** This study proposes EAT-Net (Efficient Attention Transformer Network), a dual-stream deep learning model designed to automate and enhance RCC grade classification from histopathological images. **Method:** EAT-Net integrates EfficientNetB0 for local feature extraction and a Vision Transformer (ViT) stream for capturing global contextual dependencies. The architecture incorporates Squeeze-and-Excitation (SE) modules to recalibrate feature maps, improving focus on informative regions. The model was trained and evaluated on two publicly available datasets, KMC-RENAL and RCCG-Net. Standard preprocessing was applied, and the model’s performance was assessed using accuracy, precision, recall, and F1-score. **Results:** EAT-Net achieved superior results compared to state-of-the-art models, with an accuracy of 92.25%, precision of 92.15%, recall of 92.12%, and F1-score of 92.25%. Ablation studies demonstrated the complementary value of the EfficientNet and ViT streams. Additionally, Grad-CAM visualizations confirmed that the model focuses on diagnostically relevant areas, supporting its interpretability and clinical relevance. **Conclusion:** EAT-Net offers an accurate, and explainable framework for RCC grading. Its lightweight architecture and high performance make it well-suited for clinical deployment in digital pathology workflows.

## 1. Introduction

Kidney cancer is the 14th most diagnosed cancer globally, showing gender variation in prevalence. It causes 5% of all malignancies in men, making it the sixth most diagnosed malignancy, while in women, it represents 3% of cancers and ranks as the tenth most common [1]. On the other hand, it significantly affects high-income nations, particularly in Europe and North America [2,3]. As more countries adopt Western lifestyles, the incidence of kidney cancer is expected to rise in the coming years [4].

RCC, arising from renal tubular epithelium, accounts for ~85–90% of kidney cancers [5,6]. While early treatments like IL-2 and IFN-α showed limited efficacy [7], modern approaches now include targeted therapies and immunotherapy [4,8].

The classification and understanding of RCC have significantly evolved, from the traditional categories of clear cell and granular cell carcinoma to a diverse array of histological and molecular subtypes [9,10]. The 2022 WHO classification defines ~15 RCC subtypes with distinct genetic and clinical features [11], including entities like HLRCC-associated RCC and ALK-rearranged RCC [12,13,14].

RCC grading has evolved from the 1932 Hand and Broders system to the Fuhrman and current WHO/ISUP standards, emphasizing nucleolar prominence and pleomorphism for risk stratification [15,16,17,18,19].

Manual RCC grading remains subjective, especially in intermediate cases, leading to inter-observer variability [16].

**Figure 1:**
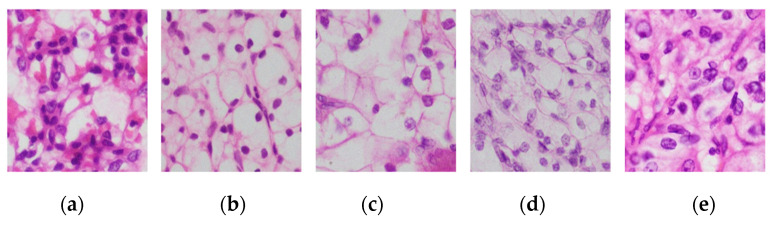
Representative H&E-Stained Images of RCC Tumor Grades. (**a**) Grade-1; (**b**) Grade-2; (**c**) Grade-3; (**d**) Grade-4; (**e**) Undifferentiated RCC.

Figure 1 exemplify RCC grades. The Figure 1a shows an example of grade 1; tumor cells have nucleoli that are absent or barely visible and appear basophilic under ×400 magnification. Figure 1b depicts grade 2, where the nucleoli appear visible and eosinophilic at ×400 magnification but remain inconspicuous at ×100 magnification. Likewise, Figure 1c presents grade 3, characterized by prominent eosinophilic nucleoli observable even at ×100 magnification, indicating more aggressive cellular features. Figure 1d represents grade 4; tumors show extreme nuclear pleomorphism and/or sarcomatous or rhabdoid differentiation, often with the presence of giant tumor cells. Figure 1e shows undifferentiated RCC or clinically aggressive tumors, although this is not universally standardized [17].

Despite advances in histological characterization, manual grading of RCC from histopathological slides remains a subjective and time-consuming process, often leading to inter-observer variability [18]. TNM staging may underrepresent tumor aggressiveness in certain cases, such as large tumors or those with sinus invasion [19].

Deep learning has emerged as an effective approach for diagnosing RCC, overcoming the challenges of manual histopathological assessment, including subjectivity and inconsistent interpretation as described by Ref. [20]. CNNs can extract complex features from tissue images with high accuracy, often outperforming traditional methods, and offer scalable, consistent diagnostics, especially in low-resource settings [21,22].

This study proposes EAT-Net, a dual-stream deep learning model for RCC grading from histopathological images. The first stream uses EfficientNetB0 for spatial feature extraction, valued for its high accuracy with fewer parameters [22,23]. The second branch incorporates a Vision Transformer (ViT) that captures global contextual features through self-attention mechanisms, demonstrating a strong performance even when annotated data are scarce [24,25]. Moreover, ViTs demonstrate robust performance even with limited labeled data, making them well-suited for medical imaging applications, where annotated datasets are often scarce [25]. The architecture balances accuracy and computational efficiency by maintaining a moderate parameter count.

- EAT-Net directly processes raw H&E-stained slides without complex preprocessing and achieves a robust performance across RCC grades 1 to 4. Its dual-stream design, enhanced by SE modules, adaptively emphasizes key features, supporting precise and reliable classification.

The remainder of this paper is organized as follows: Section 2 details the methodology, Section 3 presents experimental results, Section 4 offers discussion, and Section 5 concludes with key insights.

Accurate and consistent RCC grading is essential for guiding clinical decisions, yet manual methods are time-consuming and prone to variability. EAT-Net addresses this challenge by offering a lightweight, dual-stream deep learning solution that integrates both local and global feature understanding. Its strong performance, efficiency, and interpretability make it well-suited for real-world clinical settings, where fast, objective, and reliable grading is increasingly needed.

## 2. Proposed Methodology

### 2.1. Proposed Methods Overview

Grading RCC from histopathological images is essential for accurate prognosis and treatment planning [26]. To automate and enhance this process, we propose a hybrid A deep learning framework named EAT-Net. The proposed model merges the advantages of convolutional neural networks and context-aware transformers by coupling EfficientNetB0’s local feature extraction ability with the global contextual modeling provided by a tailored ViT stream.

Unlike conventional ViTs, the proposed transformer architecture is specifically adapted for histopathological image analysis, incorporating optimized patch embedding and attention configurations. Using self-attention mechanisms, the ViT branch learns global relationships throughout the entire image, enhancing the recognition of intricate tissue structures and subtle morphological differences among various RCC grades [27].

**Figure 2:**
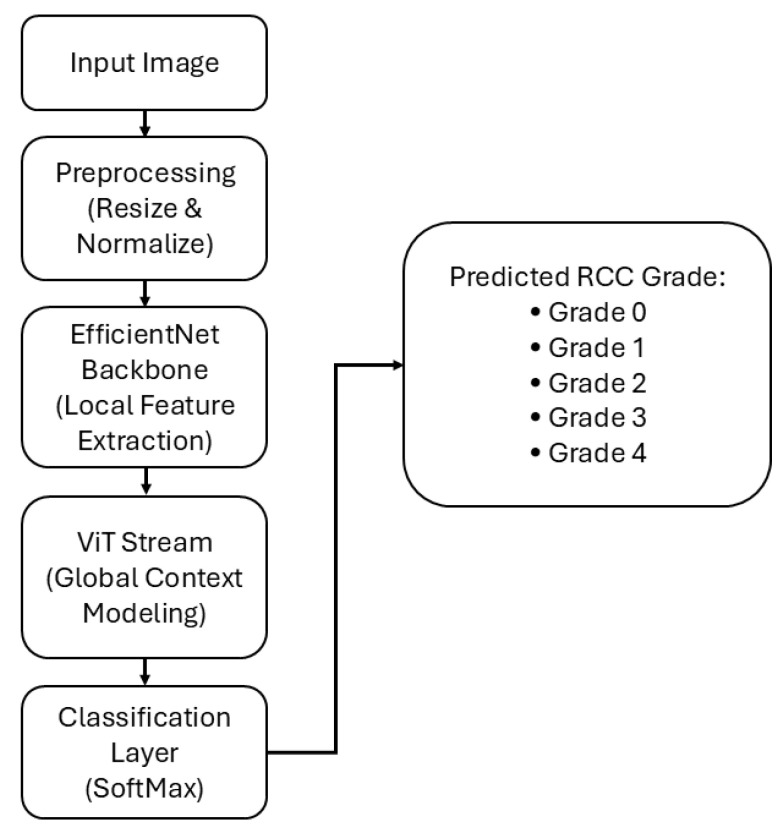
Proposed method overview diagram.

The proposed EAT-Net pipeline is illustrated in Figure 2. The process begins by resizing histopathological images to 224 × 224 pixels and applying normalization. The preprocessed images are subsequently fed into the EfficientNetB0 backbone, which derives detailed local spatial representations.

The extracted features are enhanced by SE mechanisms, which recalibrate channel importance to strengthen discriminative feature learning.

In parallel, a customized ViT stream models global contextual relationships across the image. The ViT employs self-attention mechanisms to capture long-range dependencies between spatial regions, improving the model’s understanding of complex tissue structures and subtle morphological variations.

The feature vectors from both streams are concatenated and passed through a single fully connected layer to jointly learn fused representations for RCC grade prediction. Finally, a softmax layer converts the logits into class probabilities corresponding to Grade 0–Grade 4, yielding the predicted RCC grade. This hybrid architecture enhances the model’s capacity to effectively integrate both fine-grained local details and broader contextual structures, while maintaining computational efficiency.

### 2.2. Design Principles and Architecture of EAT-Net

Microscopic histopathological images of RCC often exhibit significant variability, low contrast, and subtle morphological differences between cancerous and non-cancerous tissues, as well as among different RCC grades. Traditional CNNs, typically used for general image classification tasks, frequently struggle to capture these minor yet crucial differences accurately. This is primarily due to their general-purpose architecture, which is not optimized for the nuances of histopathological imagery [25].

To effectively address these challenges, recent research proposes tailored deep learning frameworks specifically optimized for the histopathological analysis of RCC. These frameworks follow several critical design principles.

Network depth correlates directly with computational complexity and the number of parameters. An optimal design should balance network depth to minimize the risk of overfitting while maintaining computational efficiency. EfficientNetB0, used in EAT-Net for local feature extraction, exemplifies this balance by achieving high accuracy in RCC detection and grading with significantly fewer parameters [28,29]. The ViT stream in EAT-Net captures global contextual information by modeling long-range dependencies across the image, which enhances diagnostic accuracy in classification tasks, as demonstrated in previous work on melanoma detection [30]. EfficientNetB0 is used to extract local spatial features, while the ViT stream focuses on global contextual features. The novelty lies in the customized tiny ViT that we specifically designed to extract valuable global features without consuming many parameters. This differs from generic Vision Transformers that consume approximately 86 million trainable parameters, while our ViT integrates an attention mechanism that allows the model to focus on the most discriminative regions of histopathological images and suppress irrelevant background information using 4 million trainable parameters for this stream. This enhances feature interpretability and improves grading accuracy by ensuring that the network prioritizes diagnostically relevant tissue patterns. The two streams are integrated in a lightweight dual-stream setup, enabling joint training and efficient fusion of local and global features. This hybrid design allows EAT-Net to maintain both performance and efficiency, making it a reliable framework for multi-grade RCC classification.

EAT-Net incorporates SE modules within the MBConv blocks of the EfficientNetB0 stream. These modules perform channel-wise feature recalibration by applying global average pooling followed by two fully connected layers with ReLU and Sigmoid activations. This mechanism allows the network to selectively emphasize informative channels while suppressing less relevant features, improving its ability to capture subtle morphological variations in RCC grading [31]. The proposed architecture employs an attention mechanism within the ViT stream, enabling the model to emphasize diagnostically relevant tissue regions and suppress background noise, which enhances interpretability and grading accuracy. Since both streams operate in parallel, there is no need to use separate fully connected layers for each. Instead, the feature vectors from EfficientNetB0 and the ViT stream are concatenated and passed through a single fully connected layer. This allows the network to jointly learn fused representations while maintaining a lightweight structure and reducing computational complexity. In addition, residual skip connections within each MBConv block help maintain gradient flow and support effective feature reuse during training, contributing to model stability and robustness [23]. Together, the SE modules and residual connections enhance EAT-Net’s capacity to learn discriminative features, enabling accurate classification across all five RCC grades.

**Figure 3:**
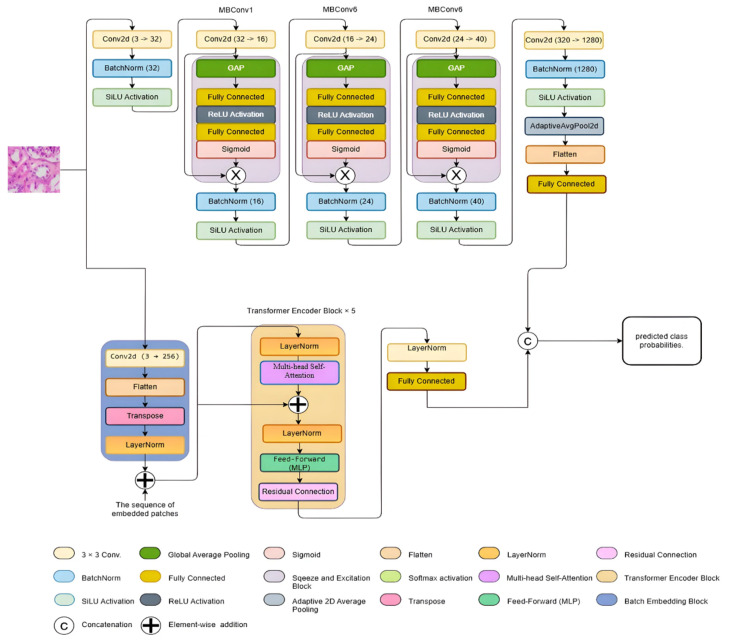
Proposed EAT-Net architecture with two stream aggregations.

The proposed EAT-Net architecture is designed as a dual-stream model, integrating the parallel fusion of two distinct components: the EfficientNetB0 stream and the ViT stream. As illustrated in Figure 3, this dual-stream configuration processes input features simultaneously through both pathways. This parallel configuration enables the model to utilize EfficientNetB0’s ability to extract local features together with the ViT stream’s strength in capturing global contextual information, thereby improving overall performance in RCC grade classification.

In the EfficientNet Stream, the input features follow a deep hierarchical structure using a series of convolutional blocks. These blocks are combined with SE modules to capture rich multi-scale spatial features effectively [31]. This stream is particularly efficient in learning fine-grained local patterns, which is crucial for detecting subtle variations in histopathological images.

On the other hand, the ViT stream processes the input by first embedding image patches using a convolutional layer and then flattening and transposing them into a sequence format. These embeddings are subsequently processed through multiple Transformer Encoder Blocks, each comprising multi-head self-attention, layer normalization, and feed-forward components.

This stream is specifically designed to model global contextual dependencies across the entire histopathological image, allowing the network to understand complex spatial relationships and morphological patterns.

**Figure 4:**
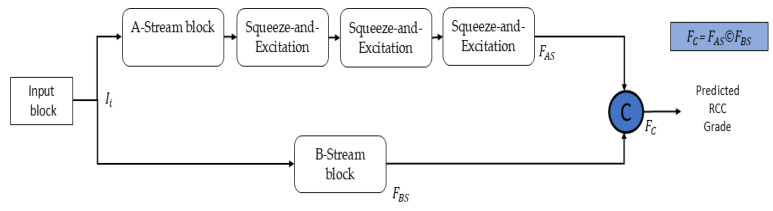
Connectivity pattern of proposed EAT-Net.

Both the EfficientNetB0-based local feature extractor and the ViT-based global context modeler independently extract high-dimensional features from the input image. These features are concatenated at the fusion point (as shown in Figure 4), forming a combined feature vector $F_{C}$, mathematically expressed in Equation (1):

*F_C_ = F_AS_ Ó F_BS_* (1)

Here, “*Ó*” represents the concatenation of features along the depth dimension, and FAS and FBS represent the output features from the EfficientNet Stream (A-Stream) and the ViT (B-Stream), respectively.

In this design, the logits from the EfficientNetB0 and ViT streams are concatenated directly rather than fused through an additional attention or gating mechanism. This choice was made to preserve the lightweight and computationally efficient nature of the model while maintaining stable training. Although attention-based or gating fusion can enhance adaptability, such mechanisms significantly increase the number of parameters and risk of overfitting, particularly when working with limited histopathological datasets. By concatenating the logits, EAT-Net effectively integrates complementary local and global representations without additional computational burden. Furthermore, the internal attention mechanism within the custom ViT stream already captures global dependencies, ensuring that the overall network benefits attention-driven contextual understanding without requiring extra fusion layers. The concatenated feature vector $F_{C}$, formed by combining the outputs of the EfficientNet and ViT streams, is not passed through an additional intermediate block or fully connected layers. Instead, each stream independently produces class predictions, and the concatenated output represents the combined logits from both classifiers. While this fusion brings together information from both the local feature extractor (EfficientNetB0) and the global context modeler (ViT), it does not involve further refinement or learning after concatenation in the current implementation.

As shown in Figure 4, the input image is simultaneously processed through two parallel pathways: the A-Stream (EfficientNetB0) and the B-Stream (Vision Transformer). Within the A-Stream, SE modules are embedded in each MBConv block to adaptively recalibrate channels by highlighting significant features while minimizing the influence of non-informative ones. This mechanism enhances the extraction of discriminative local features and strengthens model generalization with minimal computational overhead [32]. The output logits from the A-Stream (FAS) and B-Stream (FBS) are concatenated to form the final feature vector $F_{C}$, which is used to predict RCC grades. No additional fully connected layer or softmax activation is applied within the network after fusion; instead, softmax is performed externally during evaluation.

As shown in Table 1, each stream, EfficientNet and ViT, performs classification independently, producing its output logits corresponding to the five RCC grades. These logits are then concatenated, and the resulting vector is interpreted directly without passing through an additional fully connected layer. Although no softmax activation is applied within the model itself, softmax can be applied externally during evaluation to convert logits into a probability distribution over the RCC grades [33]. The proposed model is trained using the cross-entropy loss function, a widely adopted choice for multi-class classification tasks. This function measures the discrepancy between predicted logits and true class labels, guiding the optimization process to improve prediction accuracy across the five RCC grades [34]. The use of cross-entropy ensures effective and stable training by minimizing classification error.

Table 1 shows model architecture and configuration details (Input size: 224 × 224), including the number of parameters. The configuration is determined based on the resized input dimensions. Abbreviations used: Conv—Convolution layer, BN—Batch Normalization, SiLU—Sigmoid Linear Unit, GAP—Global Average Pooling, FC—Fully Connected. Concat—Feature concatenation, SE block. The EfficientNetB0 stream extracts fine-grained local features, while the ViT stream captures global contextual information. Note: No additional FC layer is used after fusion; instead, classification logits from both streams are concatenated directly for prediction. Layers marked with * include both BN and activation functions.

## 3. Experimental Environment and Results

### 3.1. Renal Cell Carcinoma Image Dataset

The study was conducted using two open-access histopathological datasets: KMC-RENAL [35] and RCCG-Net by Ref. [36]. The KMC-RENAL dataset was primarily used for training and internal evaluation, while RCCG-Net was utilized for external testing to assess model generalization. In total, 1557 images were used for training, comprising 520 for Grade 0, 312 for Grade 1, 370 for Grade 2, 158 for Grade 3, and 197 for Grade 4. For evaluation, 337 images from KMC-RENAL and 142 images from RCCG-Net were used, each labeled according to RCC tumor grades 0 through 4. The datasets used in this study are publicly available for benchmarking. To ensure a fair comparison with previous methods, we followed the same training and testing protocol defined by the original dataset providers [36] and adopted in prior works. This consistency in data splitting allows for a direct and unbiased performance comparison between EAT-Net and existing models. Accordingly, we used the same predefined training and testing splits provided by the dataset authors rather than generating new random partitions. To prepare the data for model input, all images underwent a consistent preprocessing pipeline. This included each image with 224 × 224 pixels, normalizing pixel intensity values, and converting the images to tensor format. These transformations ensured standardized input across all samples and compatibility with the model architecture, as illustrated in Figure 2.

### 3.2. EAT-Net: Training Procedures and Experimental Framework

EAT-Net employs a dual-stream architecture specifically designed for RCC grade classification. It integrates two complementary components: an EfficientNetB0 stream, which is responsible for extracting fine-grained local spatial features, and a ViT stream, which captures global contextual information through self-attention mechanisms. The EfficientNetB0 stream is initialized with pre-trained ImageNet weights and fine-tuned on the RCC dataset to leverage transfer learning, while the ViT stream is designed to process image patch embeddings and learn long-range dependencies across the histopathological image. This hybrid design enhances the model’s ability to represent both detailed morphological patterns and high-level structural context, making it robust for multi-grade RCC classification.

The dataset was divided into three subsets: 70% for training, 20% for validation, and 10% for testing. EAT-Net was trained over 65 epochs using a batch size of 16 and an initial learning rate of 0.0001. Optimization was performed using the Adam optimizer, and categorical cross-entropy was used as the loss function due to its suitability for multi-class classification tasks like RCC grading. Training was executed on a CUDA-enabled system equipped with an Intel^®^ Core™ i7-7700HQ processor, 16 GB RAM, and GPU support to accelerate computations. The cross-entropy loss used during training is mathematically defined in Equation (1).

### 3.3. Performance Evaluation of EAT-Net

To evaluate the effectiveness of the proposed EAT-Net in classifying RCC grades from histopathological images, we employed widely used performance metrics including accuracy, precision, recall, and F1 score, which collectively provide insight into the model’s classification performance across all five RCC grades (0 to 4). To ensure an accurate comparison versus existing deep learning-based approaches, EAT-Net was evaluated on the RCCG-Net dataset, which served as an independent evaluation set. The model’s performance was assessed based on standard classification outcomes: True Positives (TPs), False Positives (FPs), True Negatives (TNs), and False Negatives (FNs), as outlined in Equations (2)–(5). These values offer a dependable measure of how well the model distinguishes between different RCC grades in histopathological data.

(2) $$Accuracy=\frac{TP+TN}{TP+TN+FP+FN}$$

(3) $$Precision=\frac{TP}{TP+FP}$$

(4) $$Recall=\frac{TP}{TP+FN}$$

(5) $$F1\text{-}Score=\frac{2\cdot TP}{2\cdot TP+FP+FN}$$

### 3.4. Ablation Study for Proposed EAT-Net

To evaluate the impact of each architectural component within the EAT-Net framework, we conducted a focused ablation study across six configurations: EfficientNetB0, EfficientNetB1, EfficientNetB2, EAT-Net (EfficientNetB0 + ViT Stream), EfficientNetB1 with ViT Stream, and EfficientNetB2 with ViT Stream.

As presented in Table 2, EAT-Net (EfficientNetB0 + ViT) yielded superior results compared to other configurations. Notably, EfficientNetB0, with an input size of 224 × 224, aligns directly with the dataset, eliminating the need for interpolation. In contrast, performance of B1 and B2 may be affected due to interpolation requirements. Furthermore, EfficientNetB0 has the fewest parameters. The comprehensive EAT-Net configuration, however, exhibited enhanced consistency across all metrics. These findings indicate that the ViT Stream enhances the model’s ability to understand image patterns and perform accurate classification while minimizing parameter usage.

**Figure 5:**
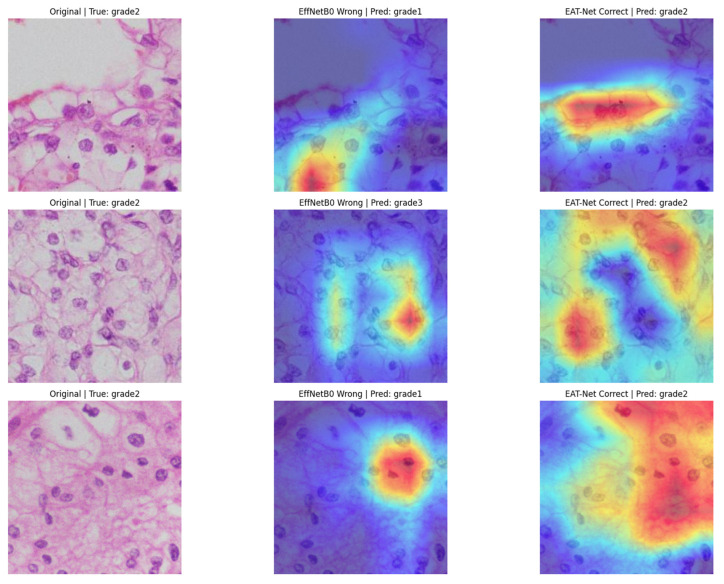
Grad-CAM visualizations comparing predictions from EfficientNetB0 and EAT-Net on RCC grade 2 samples. EAT-Net consistently attends to diagnostically relevant regions (highlighted in red and yellow), resulting in correct classifications, while EfficientNetB0 misclassifies due to scattered or misplaced focus.

To visually compare the predictive focus of each model, we generated Grad-CAM heatmaps for RCC grade 2 samples using both EfficientNetB0 and EAT-Net. As shown in Figure 5, EAT-Net consistently directed its attention to diagnostically relevant cellular regions, such as nuclei with clear morphological features. In contrast, EfficientNetB0 often misclassified the same samples by attending non-discriminative or background regions. These visualizations demonstrate that EAT-Net’s dual-stream architecture effectively captures both spatial and contextual patterns, contributing to improved classification performance

### 3.5. EAT-Net Comparison with Existing SOTA Approaches

The primary objective of this research was to create a reliable and computationally efficient deep learning model for RCC grade classification using histopathology images. To assess the effectiveness of the proposed EAT-Net architecture, we compared it with several existing deep learning models, including ResNet [37], InceptionResV2 [38], NASNet [39], ShuffleNet [40], BHCNet [41], BreastNet [42], LiverNet [43], ViT [24], RCCGNet [36], RCG-Net [26] and EFF-Net [44].

As presented in Table 3, EAT-Net outperformed all compared models across the four key evaluation metrics: Accuracy (92.58%), Precision (92.15%), Recall (92.12%), and F1-score (92.25%). These results demonstrate EAT-Net’s ability not only to match but also to surpass the performance of leading models evaluated on the KMC-RENAL dataset.

EAT-Net’s high classification performance can be attributed to its dual-stream configuration, which integrates the EfficientNetB0 backbone for spatial feature extraction with the ViT Stream. Additionally, the incorporation of SE blocks enhances channel-wise feature recalibration, while the lightweight structure of ViT Stream ensures efficient computation and faster convergence.

This consistently high performance indicates that EAT-Net can serve as a reliable model for RCC grade prediction and has the potential for application in other histopathological classification tasks. Future work may further explore its generalizability across additional datasets and its integration into clinical workflows.

## 4. Discussion

This study set out to develop a deep learning model for RCC grade classification from histopathological images. EAT-Net performed well on both the KMC-RENAL and RCCG-Net datasets, achieving 92.25% accuracy, 92.15% precision, 92.12% recall, and 92.25% F1-score with a per-image inference time of 17.95 milliseconds and 1.99 GFLOPS. These results exceeded the performance of several existing models. Grad-CAM visualizations showed that the model’s predictions aligned with key histological features, and ablation studies confirmed that combining both streams improved performance. Together, these findings support the use of EAT-Net as a dependable tool for RCC grading.

The accurate grading of RCC from histopathological images is critical for guiding prognosis and therapeutic decision-making. However, traditional approaches rely on manual interpretation, which is time-consuming, subjective, and prone to differences in judgment between pathologists [45]. To overcome these limitations, EAT-Net leverages a hybrid structure that combines convolutional efficiency with transformer-based global awareness. By integrating local features analysis and broader contextual understanding, the model delivers more consistent and reliable predictions across all RCC grades. This dual-path strategy aligns with recent findings by [37], who demonstrated that combining global and local feature maps enhances The model’s precision in identifying fine morphological distinctions in RCC grades. Local features, such as nuclear shape and texture, provide fine-grained detail, while global contextual cues help capture spatial arrangements and tissue architecture; both are essential for accurate classification. The primary objective of this study was to develop a lightweight, interpretable, and accurate deep learning framework for RCC grading, and EAT-Net achieved this by delivering high classification performance across five tumor grades while maintaining computational efficiency.

The integration of SE blocks within EAT-Net’s dual-stream architecture enhances the network’s ability to recalibrate channel-wise attention, allowing it to emphasize informative features and suppress irrelevant noise. Similar findings were reported in RenalNet, where a specialized SE-based MCRT module significantly improved classification performance with minimal added complexity. Their ablation study demonstrated a 3.54% and 3.64% drop in accuracy and F1-score, respectively, upon removing this module, underscoring the importance of attention mechanisms in RCC classification tasks [45].

Compared to RCG-Net, which uses ResNet50 with dual attention and multi-scale fusion, EAT-Net adopts a more lightweight and efficient dual-stream structure. While RCG-Net incorporates deeper residual layers and complex fusion blocks, EAT-Net avoids heavy post-fusion operations by directly combining logits from EfficientNetB0 and ViT streams. This contributes to reduced computational complexity and faster inference times, making it practical for integration into clinical workflows, especially in resource-limited environments. However, unlike RCG-Net, our model does not yet incorporate multi-scale attention mechanisms, which could be explored in future iterations [26].

EAT-Net shares key similarities with the original ViT, particularly in its ability to capture long-range dependencies through self-attention. This global contextual understanding complements the fine-grained spatial detail extracted by the Efficient Net stream, allowing EAT-Net to recognize complex patterns across RCC grades accurately. This hybrid advantage is especially critical for RCC grading, where morphological cues may be subtle and dispersed [24].

**Figure 6:**
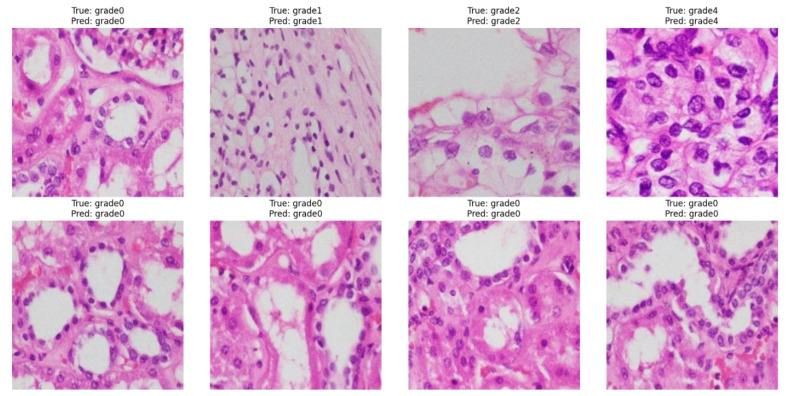
Example predictions by EAT-Net across RCC grades 0–4. All samples shown were correctly classified, with predicted grades matching the true labels.

EAT-Net’s predictions are visually interpretable, as shown in Figure 6, which displays correctly classified histopathology images across RCC grades. The first row presents accurate predictions from grades 0 to 4, highlighting the model’s ability to distinguish between subtle morphological differences, from uniform nuclei in low-grade tumors to pleomorphic structures in higher grades. The second row shows consistent predictions for multiple grade 0 cases, reflecting EAT-Net’s robustness in identifying well-differentiated tissues. This supports the model’s ability to consistently identify critical histological features important for RCC grading.

The Grad-CAM visualizations reinforce the interpretability of EAT-Net and its potential for clinical adoption. By correctly focusing on histologically meaningful areas, EAT-Net provides transparency in its decision-making process, which is essential for trust in AI-assisted pathology. Compared to EfficientNetB0, which displayed inconsistent attention in misclassified cases, EAT-Net’s focus areas closely align with those used by pathologists in grading RCC, offering further validation for its accuracy and reliability.

EAT-Net is not designed to replace pathologists, but to assist them through a collaborative, AI-enhanced workflow. With a classification accuracy of 92.25% and strong generalization to external datasets, EAT-Net offers consistent and reliable predictions across RCC grades. The model’s output is made interpretable through Grad-CAM visualizations, which highlight key histological features used in grading, helping clinicians validate its decisions. This transparency, combined with its performance, supports its use in real-world pathology settings. By reducing the time required for manual review and offering trustworthy outputs, EAT-Net can accelerate diagnosis and treatment planning, ultimately leading to more timely interventions and improved patient care.

While EAT-Net incorporates Grad-CAM to enhance interpretability, offering visual justification for its predictions, further work is needed to align with emerging regulatory standards for explainable AI in clinical diagnostics. Future extensions may explore formal explainability frameworks like SHAP or LIME to strengthen its readiness for diagnostic approval and clinical deployment.

Despite its strengths, our study has several limitations. EAT-Net was trained and tested on two publicly available datasets, which may not fully reflect real-world data diversity. These datasets primarily include clear cell and papillary RCC subtypes, which limits the model’s generalizability to other histological types based on the feature; however, the minor edits to the architecture may benefit the performance on other histopathological applications. Additionally, the use of publicly available data may introduce biases such as sampling bias, where dataset demographics may not represent the broader population, as well as labeling bias from inconsistencies in tumor annotations. Class imbalance may also reduce the model’s sensitivity to aggressive, high-grade tumors.

To reduce some of these concerns, we conducted ablation studies to validate the contribution of each model component and applied Grad-CAM visualizations to enhance interpretability. However, future work should focus on external validation using large, multi-institutional datasets with diverse histopathologists to address the potential bias. Moreover, further research should consider incorporating molecular features in line with recent WHO recommendations and expert feedback.

## 5. Conclusions

This study proposed EAT-Net, a novel dual-stream deep learning framework designed to automate the grading of RCC using histopathological images. The model integrates EfficientNetB0 for spatial feature extraction with a ViT stream for contextual understanding. Unlike conventional architectures that rely on dense fusion layers, EAT-Net directly merges logits from both streams, achieving a high diagnostic performance with reduced computational overhead. EAT-Net achieved state-of-the-art performance on the KMC-RENAL dataset, with an accuracy of 92.58% and an F1-score of 92.25%, outperforming existing deep learning models, including ResNet, ViT, and RCCG-Net. Cross-dataset testing on RCCG-Net confirmed EAT-Net’s generalizability, demonstrating robust and reliable predictions across all RCC grades. Grad-CAM visualizations demonstrated that EAT-Net focuses on relevant histological features during inference, enhancing its interpretability and alignment with clinical expectations. With its lightweight architecture, low parameter count, and elimination of computationally expensive fusion layers, EAT-Net is well-suited for real-time deployment, even in resource-constrained clinical environments. Comparative analysis of inference speed, model size, and classification accuracy further emphasizes EAT-Net’s practicality for integration into digital pathology workflows. Future research should explore applying EAT-Net to other cancers, to enhance its efficiency, and improve interpretability for real-world clinical use.

## Figures and Tables

**Table 1:** Model Architecture and Configuration Details. K denotes Kernel size, F denotes Filters, O/P denotes output, and Pram denotes the number of parameters.

Stream	Name (Layer)	K	Stride	F	O/P Size	Param.
Efficient Stream	Conv2d (3 → 32)	3 × 3	1	32	224 × 224 × 32	896
	BatchNorm	-	-	32	224 × 224 × 32	128
	SiLU Activation	-	-	-	-	-
	MBConv1-GAP (AvgPool)	-	-	-	1 × 1 × 16	-
	MBConv1-FC1	1 × 1	-	256	1 × 1 × 256	256
	MBConv1-ReLU	-	-	-	-	-
	MBConv1-FC2	1 × 1	-	16	1 × 1 × 16	256
	MBConv1-Sigmoid *	-	-	-	-	-
	Conv2d (32 → 16) *	3 × 3	1	16	224 × 224 × 16	4608
	MBConv6–GAP (AvgPool)	-	-	-	1 × 1 × 40	-
	Conv2d (24 → 40) *	3 × 3	1	40	112 × 112 × 40	21,600
	Flatten	-	-	-	-	-
ViT Stream	Conv2d (3 → 256) * (Patch Embed)	16 × 16	16	256	14 × 14 × 256	18432
	Flatten	-	-	-	1 × 196 × 256	-
	Transpose	-	-	-	196 × 256	-
	Transformer Encoder ×5	-	-	256 (per head)	196 × 256	≈786,000
	LayerNorm + FC	-	-	256 → Num_classes	1 × 256	65,792
Final	Fusion-Concatenate	-	-	512 (256 + 256)	1 × 512	-
	Predicted Output (Logits)	-	-	Num_classes	Num_classes	-

**Table 2:** EAT-Net ablation study.

Method	Parameters (Millions)	Accuracy	Precision	Recall	F1-Score
EfficientNetB0 only	4	92.96	91.57	91.56	91.57
EfficientNetB1 only	7.8	91.55	90.12	90.05	90.08
EfficientNetB2 only	9.2	90.14	88.48	88.30	88.39
EfficientNetB1 + ViT Stream	15.9	92.25	91.46	91.15	91.30
EfficientNetB2 + ViT Stream	17.3	90.85	90.95	90.83	90.89
EAT-Net (EfficientNetB0 + ViT Stream)	12.1	92.25	92.15	92.12	92.25

**Table 3:** Comparative performance analysis of the proposed EAT-Net against current state-of-the-art models for RCC grade classification.

Method	Parameters (Million)	GFLOPS	Accuracy	Precision	Recall	F1-Score
ResNet [37]	23.60	7.75	74.64	73.60	70.97	70.93
IncResV2 [38]	54.34	13	71.83	71.85	68.64	68.69
NASNet [39]	4.27	1.15	80.28	79.69	77.99	77.09
ShuffleNet [40]	23.83	6.69	83.80	83.15	81.75	81.86
BHCNet [41]	0.30	4.46	86.61	85.08	84.62	84.34
BreastNet [42]	0.61	5.68	85.91	86.88	85.47	85.41
LiverNet [43]	0.57	3.72	86.62	85.52	84.69	84.93
ViT [24]	86.00	33.03	82.39	81.60	80.88	80.38
RCCGNet [36]	0.36	4.48	90.14	89.78	89.60	89.06
RCG-Net [26]	2.38	1.68	90.62	91.23	90.63	90.92
EFF-Net [44]	-	-	91.90	91.42	91.84	91.90
EAT-Net (Proposed)	12.1	1.99	92.25	92.15	92.12	92.25

## Data Availability

There is no new data generated in this study. The publicly available dataset mentioned in Section 3.1 can be accessed via this link: https://www.kaggle.com/datasets/shreyan983/kmc-renal (accessed on 31 October 2025).

## References

[1] Rose T.L., Kim W.Y. (2024). Renal Cell Carcinoma: A Review. JAMA.

[2] Wong M.C.S., Goggins W.B., Yip B.H.K., Fung F.D.H., Leung C., Fang Y., Wong S.Y.S., Ng C.F. (2017). Incidence and Mortality of Kidney Cancer: Temporal Patterns and Global Trends in 39 Countries. Sci. Rep..

[3] Mathew A., Devesa S.S., Fraumeni J.F.J., Chow W.-H. (2002). Global Increases in Kidney Cancer Incidence, 1973–1992. Eur. J. Cancer Prev..

[4] Makino T., Kadomoto S., Izumi K., Mizokami A. (2022). Epidemiology and Prevention of Renal Cell Carcinoma. Cancers.

[5] Hsieh J.J., Purdue M.P., Signoretti S., Swanton C., Albiges L., Schmidinger M., Heng D.Y., Larkin J., Ficarra V. (2017). Renal Cell Carcinoma. Nat. Rev. Dis. Primers.

[6] Young M., Jackson-Spence F., Beltran L., Day E., Suarez C., Bex A., Powles T., Szabados B. (2024). Renal Cell Carcinoma. Lancet.

[7] Johannsen M., Brinkmann O.A., Bergmann L., Heinzer H., Steiner T., Ringsdorf M., Römer A., Roigas J. (2007). The Role of Cytokine Therapy in Metastatic Renal Cell Cancer. Eur. Urol. Suppl..

[8] Yang S., Yang X., Hou Z., Zhu L., Yao Z., Zhang Y., Chen Y., Teng J., Fang C., Chen S. (2024). Rationale for Immune Checkpoint Inhibitors plus Targeted Therapy for Advanced Renal Cell Carcinoma. Heliyon.

[9] Wang H.-Y., Mills S.E. (2005). KIT and RCC Are Useful in Distinguishing Chromophobe Renal Cell Carcinoma From the Granular Variant of Clear Cell Renal Cell Carcinoma. Am. J. Surg. Pathol..

[10] Saleeb R.M., Brimo F., Farag M., Rompré-Brodeur A., Rotondo F., Beharry V., Wala S., Plant P., Downes M.R., Pace K. (2017). Toward Biological Subtyping of Papillary Renal Cell Carcinoma With Clinical Implications Through Histologic, Immunohistochemical, and Molecular Analysis. Am. J. Surg. Pathol..

[11] Warren A.Y., Harrison D. (2018). WHO/ISUP Classification, Grading and Pathological Staging of Renal Cell Carcinoma: Standards and Controversies. World J. Urol..

[12] Mercken K., Berkel B.V., Wever L.D. (2024). Hereditary Leiomyomatosis and Renal Cell Cancer (HLRCC) Syndrome. J. Belg. Soc. Radiol..

[13] Kumar R., Bonert M., Naqvi A., Zbuk K., Kapoor A. (2018). SDH-Deficient Renal Cell Carcinoma—Clinical, Pathologic and Genetic Correlates: A Case Report. BMC Urol..

[14] Galea L.A., Hildebrand M.S., Witkowski T., Joy C., McEvoy C.R., Hanegbi U., Aga A. (2023). ALK-Rearranged Renal Cell Carcinoma with TPM3::ALK Gene Fusion and Review of the Literature. Virchows Arch..

[15] Oki R., Takemura K., Urasaki T., Fujiwara R., Numao N., Yonese J., Miura Y., Yuasa T. (2025). Prevailing Challenges in Personalized Treatment for Metastatic Renal Cell Carcinoma: A Narrative Review. Expert. Rev. Anticancer. Ther..

[16] Khene Z.-E., Kammerer-Jacquet S.-F., Bigot P., Rabilloud N., Albiges L., Margulis V., De Crevoisier R., Acosta O., Rioux-Leclercq N., Lotan Y. (2024). Clinical Application of Digital and Computational Pathology in Renal Cell Carcinoma: A Systematic Review. Eur. Urol. Oncol..

[17] Nezami B.G., MacLennan G.T. (2025). Clear Cell Renal Cell Carcinoma: A Comprehensive Review of Its Histopathology, Genetics, and Differential Diagnosis. Int. J. Surg. Pathol..

[18] Mardi L., Tauziède-Espariat A., Guillemot D., Pierron G., Gigant P., Mehdi L., Berthaud C., Pucelle N., Lacombe J., Hasty L. (2021). BCOR Immunohistochemistry, but Not SATB2 Immunohistochemistry, Is a Sensitive and Specific Diagnostic Biomarker for Central Nervous System Tumours with BCOR Internal Tandem Duplication. Histopathology.

[19] Dai Y., Hu W., Wu G., Wu D., Zhu M., Luo Y., Wang J., Zhou Y., Hu P. (2024). Grading Clear Cell Renal Cell Carcinoma Grade Using Diffusion Relaxation Correlated MR Spectroscopic Imaging. J. Magn. Reson. Imaging.

[20] Zhu M., Ren B., Richards R., Suriawinata M., Tomita N., Hassanpour S. (2021). Development and Evaluation of a Deep Neural Network for Histologic Classification of Renal Cell Carcinoma on Biopsy and Surgical Resection Slides. Sci. Rep..

[21] Tabibu S., Vinod P.K., Jawahar C.V. (2019). Pan-Renal Cell Carcinoma Classification and Survival Prediction from Histopathology Images Using Deep Learning. Sci. Rep..

[22] Unger M., Kather J.N. (2024). A Systematic Analysis of Deep Learning in Genomics and Histopathology for Precision Oncology. BMC Med. Genom..

[23] Zhou T., Ye X., Lu H., Zheng X., Qiu S., Liu Y. (2022). Dense Convolutional Network and Its Application in Medical Image Analysis. BioMed Res. Int..

[24] Dosovitskiy A., Beyer L., Kolesnikov A., Weissenborn D., Zhai X., Unterthiner T., Dehghani M., Minderer M., Heigold G., Gelly S. (2021). An Image Is Worth 16x16 Words: Transformers for Image Recognition at Scale. arXiv.

[25] Wang X., Yang S., Zhang J., Wang M., Zhang J., Huang J., Yang W., Han X., de Bruijne M., Cattin P.C., Cotin S., Padoy N., Speidel S., Zheng Y., Essert C. (2021). TransPath: Transformer-Based Self-Supervised Learning for Histopathological Image Classification. Proceedings of the Medical Image Computing and Computer Assisted Intervention—MICCAI 2021.

[26] Mahmood T., Wahid A., Hong J.S., Kim S.G., Park K.R. (2024). A Novel Convolution Transformer-Based Network for Histopathology-Image Classification Using Adaptive Convolution and Dynamic Attention. Eng. Appl. Artif. Intell..

[27] Zhang Y., Wang J., Gorriz J.M., Wang S. (2023). Deep Learning and Vision Transformer for Medical Image Analysis. J. Imaging.

[28] Shah H.A., Saeed F., Yun S., Park J.-H., Paul A., Kang J.-M. (2022). A Robust Approach for Brain Tumor Detection in Magnetic Resonance Images Using Finetuned EfficientNet. IEEE Access.

[29] Kumar Y., Brar T.P.S., Kaur C., Singh C. (2024). A Comprehensive Study of Deep Learning Methods for Kidney Tumor, Cyst, and Stone Diagnostics and Detection Using CT Images. Arch. Comput. Methods Eng..

[30] Aksoy S., Demircioglu P., Bogrekci I. (2024). Enhancing Melanoma Diagnosis with Advanced Deep Learning Models Focusing on Vision Transformer, Swin Transformer, and ConvNeXt. Dermatopathology.

[31] Mpofu J.B., Li C., Gao X., Su X. (2024). Optimizing Motion Detection Performance: Harnessing the Power of Squeeze and Excitation Modules. PLoS ONE.

[32] Zeng C., Zhao Y., Wang Z., Li K., Wan X., Liu M. (2025). Squeeze-and-Excitation Self-Attention Mechanism Enhanced Digital Audio Source Recognition Based on Transfer Learning. Circuits Syst. Signal Process.

[33] Dubey S.R., Singh S.K., Chaudhuri B.B. (2022). Activation Functions in Deep Learning: A Comprehensive Survey and Benchmark. Neurocomputing.

[34] Farhadpour S., Warner T.A., Maxwell A.E. (2024). Selecting and Interpreting Multiclass Loss and Accuracy Assessment Metrics for Classifications with Class Imbalance: Guidance and Best Practices. Remote Sens..

[35] KMC-RENAL. https://www.kaggle.com/datasets/shreyan983/kmc-renal.

[36] Chanchal A.K., Lal S., Kumar R., Kwak J.T., Kini J. (2023). A Novel Dataset and Efficient Deep Learning Framework for Automated Grading of Renal Cell Carcinoma from Kidney Histopathology Images. Sci. Rep..

[37] Shafiq M., Gu Z. (2022). Deep Residual Learning for Image Recognition: A Survey. Appl. Sci..

[38] Szegedy C., Ioffe S., Vanhoucke V., Alemi A. Inception-v4, Inception-ResNet and the Impact of Residual Connections on Learning. Proceedings of the AAAI Conference on Artificial Intelligence.

[39] Zoph B., Vasudevan V., Shlens J., Le Q.V. Learning Transferable Architectures for Scalable Image Recognition. Proceedings of the 2018 IEEE/CVF Conference on Computer Vision and Pattern Recognition.

[40] Zhang X., Zhou X., Lin M., Sun J. ShuffleNet: An Extremely Efficient Convolutional Neural Network for Mobile Devices. Proceedings of the 2018 IEEE/CVF Conference on Computer Vision and Pattern Recognition.

[41] Jiang Y., Chen L., Zhang H., Xiao X. (2019). Breast Cancer Histopathological Image Classification Using Convolutional Neural Networks with Small SE-ResNet Module. PLoS ONE.

[42] Toğaçar M., Özkurt K.B., Ergen B., Cömert Z. (2020). BreastNet: A Novel Convolutional Neural Network Model through Histopathological Images for the Diagnosis of Breast Cancer. Phys. A Stat. Mech. Its Appl..

[43] Aatresh A.A., Alabhya K., Lal S., Kini J., Saxena P.P. (2021). LiverNet: Efficient and Robust Deep Learning Model for Automatic Diagnosis of Sub-Types of Liver Hepatocellular Carcinoma Cancer from H&E Stained Liver Histopathology Images. Int. J. CARS.

[44] Maqsood F., Wang Z., Ali M.M., Qiu B., Mahmood T., Sarwar R. (2024). An Efficient Enhanced Feature Framework for Grading of Renal Cell Carcinoma Using Histopathological Images. Appl. Intell..

[45] Chanchal A.K., Lal S., Suresh S. (2025). Development and Evaluation of Deep Neural Networks for the Classification of Subtypes of Renal Cell Carcinoma from Kidney Histopathology Images. Sci. Rep..

